# Temporal Dynamics of Reactive Oxygen and Nitrogen Species and NF-κB Activation During Acute and Chronic T Cell–Driven Inflammation

**DOI:** 10.1007/s11307-019-01412-8

**Published:** 2019-09-03

**Authors:** Johannes Schwenck, Roman Mehling, Wolfgang M. Thaiss, Daniela Kramer, Irene Gonzalez Menendez, Hasan Halit Öz, Dominik Hartl, Klaus Schulze-Osthoff, Stephan Hailfinger, Kamran Ghoreschi, Leticia Quintanilla-Martinez, Harald Carlsen, Martin Röcken, Bernd J. Pichler, Manfred Kneilling

**Affiliations:** 1 Werner Siemens Imaging Center, Department of Preclinical Imaging and Radiopharmacy, Eberhard Karls University, 72076 Tübingen, Germany; 2 Department of Nuclear Medicine, Eberhard Karls University, 72076 Tübingen, Germany; 3 Department of Diagnostic and Interventional Radiology, Eberhard Karls University, 72076 Tübingen, Germany; 4 Interfaculty Institute of Biochemistry, Eberhard Karls University of Tübingen, Tübingen, Germany; 5 Department of Pathology, Eberhard Karls University, 72076 Tübingen, Germany; 6 Department of Pediatrics I, Eberhard Karls University, 72076 Tübingen, Germany; 7 German Cancer Consortium (DKTK) and German Cancer Research Center, 69120 Heidelberg, Germany; 8 Department of Dermatology, Venereology and Allergology, Charité – Universitätsmedizin Berlin, 10117 Berlin, Germany; 9 Department of Chemistry, Biotechnology and Food Science, Norwegian University of Life Sciences, 1432 Ås, Norway; 10 Department of Dermatology, Eberhard Karls University, 72076 Tübingen, Germany

**Keywords:** Optical imaging, Delayed-type hypersensitivity reaction, Contact allergy, N-acetylcysteine, Contact hypersensitivity reaction, Inflammation, Anti-inflammatory effect, L-012, NF-κB

## Abstract

**Purpose:**

Reactive oxygen and nitrogen species (ROS/RNS) production and the NF-κB activation are critically involved in inflammatory responses, but knowledge about the temporal dynamics during acute and chronic inflammation is limited. Here, we present a comparative longitudinal *in vivo* study of both parameters in an experimental model of acute and chronic T cell–driven delayed-type hypersensitivity reaction (DTHR) using noninvasive optical imaging.

**Procedures:**

Trinitrochlorobenzene (TNCB)-sensitized NF-κB-luciferase-reporter and wild-type mice were TNCB challenged on the right ear to elicit acute DTHR and then repetitively challenged (up to five times) to induce chronic DTHR. Mice were treated with the ROS-scavenging and NF-κB inhibiting molecule N-acetylcysteine (NAC) or underwent sham treatment. ROS/RNS production was noninvasively analyzed *in vivo* using the ROS-/RNS-sensitive chemiluminescent probe L-012, and NF-κB activation was measured using NF-κB-luciferase-reporter mice. H&E staining, CD3 and myeloperoxidase (MPO) immunohistochemistry (IHC), and quantitative PCR (qPCR) analyses were employed to investigate immune cell infiltration and expression of NF-κB- and ROS-/RNS-driven genes.

**Results:**

In acute DTHR, we found strongly elevated ROS/RNS production and NF-κB activation 12 h after the 1st TNCB ear challenge, peaking at 24 h after the challenge. In chronic DTHR, ROS production peaked as early as 4 h after the 5th TNCB challenge, whereas NF-κB activity peaked after 12 h. The increase in ROS/RNS production in acute DTHR was higher than the increase in NF-κB activity but the relationship was inverse in chronic DTHR. Treatment with the ROS scavenger NAC had differential effects on ROS/RNS production and NF-κB activation during acute and chronic DTHR. *Ex vivo* cross-validation by histopathology and qPCR analysis correlated closely with the *in vivo* imaging results.

**Conclusions:**

Noninvasive *in vivo* imaging is capable of assessing the temporal dynamics of ROS/RNS production and NF-κB activation during progression from acute to chronic DTHR and enables monitoring of anti-inflammatory treatment responses.

**Electronic supplementary material:**

The online version of this article (10.1007/s11307-019-01412-8) contains supplementary material, which is available to authorized users.

## Introduction

Reactive oxygen species (ROS) and reactive nitrogen species (RNS), a heterogeneous group of oxidative molecules, are common stress factors for cells. ROS/RNS appear in both physiological, e.g., as byproducts of cellular metabolism or as signaling molecules, and pathological conditions, such as when ROS/RNS are generated by inflammatory cells, including polymorphonuclear neutrophils (PMNs) or macrophages [1, 2].

In particular, oxidative phosphorylation in mitochondria and NADPH oxidase complexes (NOX) convert oxygen into the primary radical superoxide anion (O_2_^−^), which is rapidly converted into secondary ROS/RNS. Membrane-permeable hydrogen peroxide (H_2_O_2_) is generated by catalase or glutathione peroxidase. Myeloperoxidase (MPO) generates various other ROS, such as hypochlorous acid (HClO), hypothiocyanous acid (HOSCN), and other radicals, by oxidation of organic and inorganic substrates [3]. Further, RNS such as peroxynitrite are formed by a reaction between O_2_^−^ and nitric oxide (NO), which is produced by nitric oxide synthases (NOS) [4]. Since ROS/RNS participate in multiple biochemical interactions, the roles of ROS/RNS in inflammation have more than one dimension: both pro- and anti-inflammatory roles have been described, which lead to either tissue-destructive or tissue-protective effects [5].

Therefore, maintenance of a regulated balance between ROS/RNS and antioxidants is necessary for the control of immune responses. The multiple effects of ROS become evident in patients with chronic granulomatous disease (CGD), which is caused by an inherited deficiency of NOX2 activity. CGD patients suffer from both persistent bacterial and fungal infections as well as autoimmune diseases such as arthritis [6].

The immunomodulatory effects of ROS/RNS on inflammatory immune responses are caused by a variety of mechanisms, including interactions with signaling pathways such as nuclear factor (erythroid-derived 2)-like 2 (NRF2) or p38 mitogen-activated protein (MAP) kinases [7], but they also influence the mechanisms of antigen presentation and T cell receptor signaling as well as aerobic glycolysis in activated CD4^+^ T cells [3]. A major ROS-sensitive regulator of inflammatory immune responses is the NF-κB pathway [8]. The NF-κB protein family consists of five proteins that can form multiple heterodimeric NF-κB protein complex and induce the transcription of many genes, including pro-inflammatory mediators such as IL-1β, IL-6, and TNF as well as other pro- and antioxidative target genes [9].

So far, little is known about the temporal dynamics of ROS/RNS production and their interactions with inflammatory pathways, such as NF-κB. Due to the short lifetime, ranging from milliseconds to minutes, and the chemical variety of different ROS, measurement of ROS/RNS remains challenging. A few detection strategies have been described to noninvasively study ROS production *in vivo* [10–17].

L-012 is a luminol-based chemiluminescent (CL) probe, which was evaluated for preclinical *in vivo* optical imaging (OI) experiments by Kielland et al. [10]. The oxidized form of L-012 reacts with O_2_^−^ to form an excited-state intermediate, which emits detectable photons by chemiluminescence [11]. Some investigators have suggested that RNS lead to a pronounced L-012 chemiluminescent signal, while the contribution of H_2_O_2_ to the luminescent signal is relatively small [11, 18].

In this study, we focused on the temporal dynamics of ROS/RNS production and their influence on NF-κB signaling by noninvasive *in vivo* OI in acute and chronic TNCB-induced cutaneous contact hypersensitivity, a well-characterized and established experimental model for T cell–driven DTHR [19–21]. DTHRs are orchestrated mainly by interferon-γ-producing CD4^+^ (Th1) and CD8^+^ (Tc1) T cells [22, 23] and characterized by accumulations of PMNs. PMNs elicit ROS/RNS during oxidative burst, which is critically involved in the pathogenesis of several autoimmune diseases, such as rheumatoid arthritis and psoriasis vulgaris [24].

To our knowledge, this is the first noninvasive *in vivo* study investigating the temporal dynamics of ROS/RNS production and NF-κB activation in parallel during different stages of inflammation, employing L-012 and NF-κB-luciferase-reporter mice combined with *ex vivo* cross-validation employing H&E staining, CD3- and MPO-IHC, and qPCR analysis of NF-κB- and ROS-driven genes. In addition, we studied the influence of NAC treatment on ROS/RNS production and NF-κB activation dynamics using these two imaging tools.

## Materials and Methods

### Animal Experiments

We used 8- to 12-week-old female C57BL/6 mice from Charles River Laboratories (Sulzfeld, Germany) and NF-κB-luciferase-reporter mice provided by Harald Carlsen (Norwegian University of Life Sciences, Ås, Norway) [25]. Animal experiments were approved by the Regierungspräsidium Tübingen. The details on the animal experiments are provided in the electronic supplementary material (ESM).

### Treatment Approach

Two days prior to the first TNCB ear challenge, we started to add NAC continuously to the drinking water until the experiments were finished (5 mg/ml; Sigma-Aldrich, Steinheim, Germany) [20]. Sham-treated mice received normal drinking water.

### Optical Imaging

To measure *in vivo* NF-κB activation, we injected luciferin (150 mg/kg body weight) i.p. into NF-κB-luciferase-reporter mice 5 min before OI (*n* = 10). For *in vivo* ROS detection, we injected wild-type mice with ROS-sensitive L-012 (25 mg/kg body weight; i.p.) 5 min before OI (*n* = 8). L-012 (Wako Chemical, Neuss, Germany) was dissolved in ultrapure H_2_O at a concentration of 5 mg/ml. To assess L-012 chemiluminescence and NF-κB-luciferase bioluminescence signals *in vivo*, we used the IVIS Spectrum OI System (PerkinElmer, Rodgau-Jügesheim, Germany). For details, see ESM.

### RNA Extraction and Gene Expression Analysis, Histopathology, and Statistical Analysis

Details are described in the ESM.

## Results

### Time Course of ROS Production and NF-κB Activity in Acute and Chronic Cutaneous DTHR

**Fig. 1:**
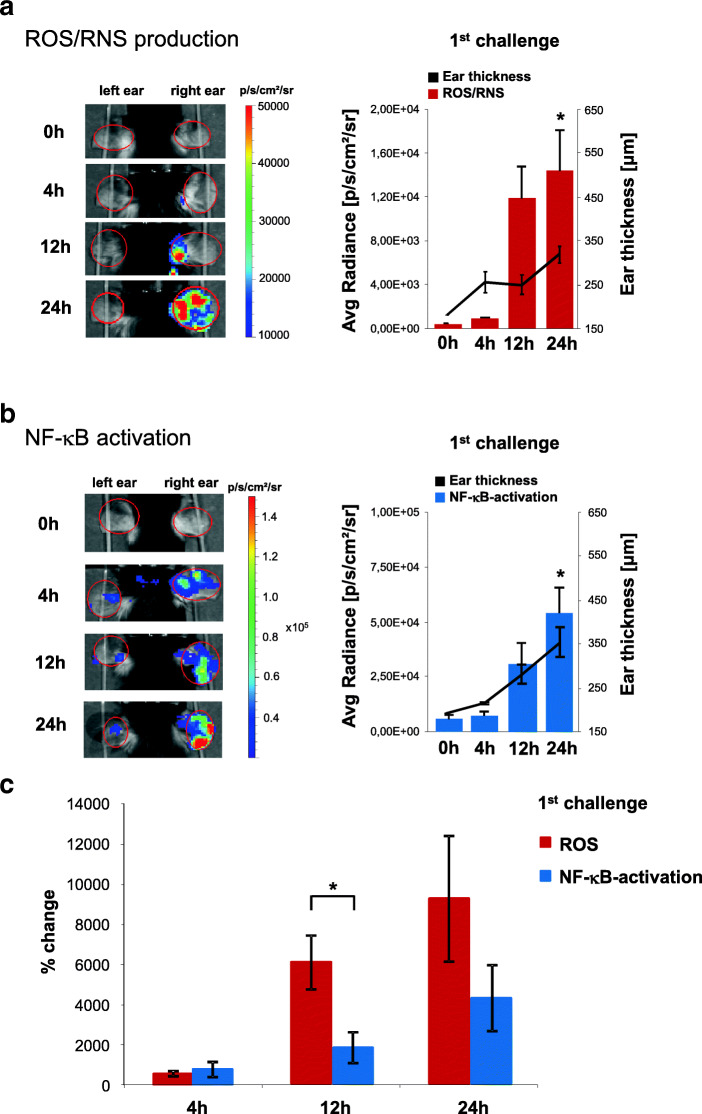
ROS/RNS production and NF-κB activity in C57BL/6 wild-type mice and C57BL/6 NF-B-luciferase-reporter mice with acute cutaneous DTHR; L-012: *n* = 8; NF-κB-luciferase-reporter mice: *n* = 10. (A) Temporal dynamics of ROS/RNS production (L-012 optical imaging). The peak of the signal intensity (24 h) and the baseline signal intensity was compared using a paired, two tailed Student’s *t* test. (B) Temporal dynamics of NF-κB activity. (NF-κB-luciferase-reporter mice) the peak of the signal intensity (24 h) and the baseline signal intensity was compared using a paired, two tailed Student’s *t* test. (C) To compare the increase in ROS/RNS production with the increase in NF-κB activation, we calculated the relative change compared with baseline (not inflamed healthy ear) at 0 h, initially before the 1st TNCB ear challenge. Relative enhancement of ROS/RNS production was significantly stronger than relative activation of NF-κB 12 h after the 1st TNCB challenge (unpaired, two-tailed Student’s *t* test). Data are presented as the mean ± SEM.

First, we evaluated the time course of ROS/RNS production and NF-κB activity in acute DTHR at baseline, 4 h, 12 h, and 24 h after the 1st challenge. At baseline, we recorded only very faint L-012 signal in ears of wild-type mice (Fig. 1a). Additionally, a faint luciferase-mediated signal was recorded in inflamed ears of NF-κB reporter mice (Fig. 1b). As early as 4 h after the 1st TNCB challenge, ear thickness had increased, but we found no enhancement of ROS/RNS production or NF-κB activation (Fig. 1a and b). However, 12 h after the 1st TNCB challenge, both ROS/RNS production and NF-κB activity in inflamed ears increased dramatically (ROS/RNS: 60-fold; NF-κB activity: 18-fold) when compared with baseline and it further increased at 24 h.

We next compared the signal intensities from L-012 chemiluminescence and bioluminescence from inflamed ears of NF-κB-reporter mice at baseline with the signal intensities at 4 h, 12 h, and 24 h post-TNCB challenge. The increase in ROS-/RNS-mediated L-012 signal intensity in inflamed ears was significantly higher than the measured increase in NF-κB activity 12 h after the first TNCB challenge (Fig. 1c).

**Fig. 2:**
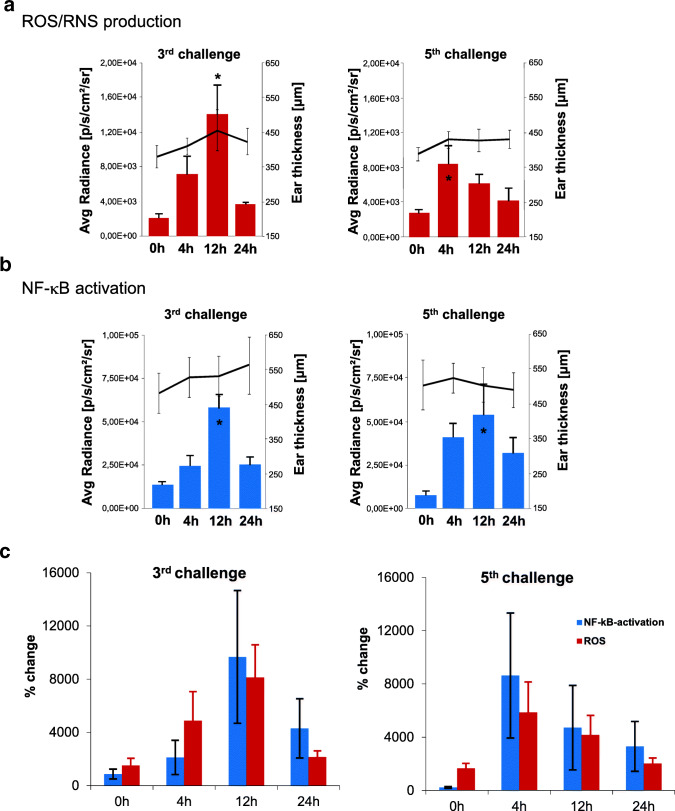
ROS/RNS production and NF-κB activity in C57BL/6 wild-type mice and C57BL/6 NF-κB-luciferase-reporter mice with early chronic and cutaneous chronic DTHR; L-012: *n* = 8; NF-κB-luciferase-reporter mice: *n* = 10. (A) Temporal dynamics of ROS/RNS production (L-012 optical imaging). The peak of the signal intensity (24 h) and the baseline signal intensity was compared using a paired, two tailed Student’s *t* test. (B) Temporal dynamics of NF-κB activity (NF-κB-luciferase-reporter mice). The peak of the signal intensity (24 h) and the baseline signal intensity was compared using a paired, two tailed Student’s *t* test. (C) To compare the increase in ROS/RNS production with the increase in NF-B activation, we calculated the relative change compared with baseline in healthy not inflamed ears initially before (0 h) the 1st TNCB ear challenge. Data are presented as the mean ± SEM.

As repeated TNCB challenges lead to chronic cutaneous DTHR [20], we investigated the time course of ROS/RNS and NF-κB activity after the 3rd and 5th TNCB ear challenges. During early chronic DTHR, we determined clear enhancement of ROS/RNS production and NF-κB activity in inflamed ears by 4 h after the 3rd TNCB ear challenge (Fig. 2a and b). The peak in ROS/RNS production and NF-κB activity shifted from 24 h after the 1st TNCB ear challenge to 12 h after the 3rd TNCB ear challenge. In contrast, the ear thickness indicated no significant change between 12 h and 24 h after the 3rd TNCB ear challenge. ROS/RNS production was already present as early as 4 h after the 5th TNCB ear challenge, whereas the NF-κB activity did not peak until 12 h after the 5th challenge (Fig. 2a and b).

**Fig. 3:**
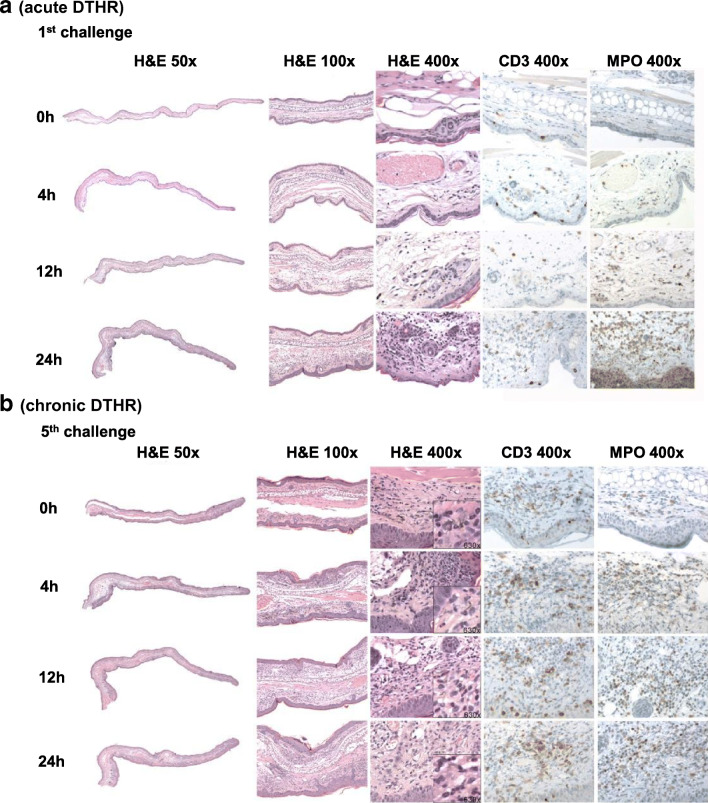
Standard H&E histopathology as well as CD3 and MPO IHC from (a) naïve mice (0 h), inflamed ears with (a) acute cutaneous DTHR 4 h, 12 h, and 24 h after the 1st TNCB ear challenge and (b) chronic cutaneous DTHR before (48 h after the 4th TNCB ear challenge) 4 h, 12 h, and 24 h after the 5th TNCB ear challenge (*n* = 4; only 12 h after the 5th TNCB ear challenge: *n* = 3).

When comparing the relative change in ROS/RNS production and NF-κB activity in the inflamed ears with early chronic and chronic cutaneous DTHR to the baseline at 0 h before the 1st TNCB ear challenge, we determined before (0 h) the 3rd and 5th TNCB ear challenge slightly elevated NF-κB activity values. ROS/RNS production values were remarkably elevated most probably due to the PNMs infiltrating the chronically inflamed ears after the previous TNCB challenges (Figs. 2c, 3b). The relative increase in NF-κB activity at 12 h and 24 h after the 3rd and 4 h, 12 h, and 24 h after the 5th TNCB ear challenge was more pronounced than the increase in ROS/RNS production (Fig. 2c). Interestingly, when calculating the relative change in signal intensity within the course of the 3rd or 5th TNCB ear challenge, using the 0 - h timepoint of the 3rd or 5th challenge as baseline, the relative increase in NF-κB activity 12 h and 48 h after the 3rd TNCB ear challenge and especially 4 h, 12 h, and 24 h after the 5th TNCB ear challenge rose up to 80-fold while the relative increase in ROS/RNS production was moderate (Suppl. Fig. 1, see ESM).

### *Ex vivo* Analysis of Inflamed Ears with Acute and Chronic Cutaneous DTHR

For *ex vivo* cross-validation of our *in vivo* imaging results on ROS/RNS production and NF-κB activity, we performed H&E staining and CD3- and MPO-IHC as well as qPCR analysis focusing on NF-κB and ROS-/RNS-driven genes in inflamed ears with acute and chronic cutaneous DTHR.

Standard H&E histopathology as well as CD3- and MPO-IHC revealed in inflamed ears with acute cutaneous DTHR (4 h after the 1st TNCB ear challenge) edema with dilated blood vessels and the presence of MPO^+^ PMNs within the blood vessels, but not in the surrounding tissue. Only scarce CD3^+^ T cells were found in the dermis. At 12 h after the 1st TNCB ear challenge, the edema was more evident, with large dilated blood vessels and extravasation of PMNs, which were also found in large clusters in the dermis. Scarce CD3^+^ T cells and macrophages were present within the epidermis and in the dermis, while PMNs were significantly increased in the dermis. Strikingly, 24 h after the 1st TNCB ear challenge, a massive infiltration of PMNs within the dermis accompanied with abscesses and small crusts was observed together with a mild increase of CD3^+^ T cells and macrophages within the dermis **(**Fig. 3a; Suppl. Table 2; Suppl. Fig. 2—see ESM).

Before the 5th TNCB ear challenge, 48 h after the 4th TNCB ear challenge, we observed thickening of the epidermis as a consequence of the chronic inflammation. The dermis showed a mild edema accompanied with dilated blood vessels and an infiltrate of CD3^+^ T cells and macrophages but only a few MPO^+^ PMNs. After 4 h, 12 h, and 24 h, a similar thickened epidermis with few small crusts and abscesses were identified together with an increased infiltrate of CD3^+^ T cells and MPO^+^ PMNs within the dermis (Fig. 3b; Suppl. Table 2; Suppl. Fig. 2—see ESM). In summary, the histopathological evaluation of the degree of inflammation and the homing dynamics of MPO^+^ PMNs during acute and chronic cutaneous DTHR fit well with our *in vivo* ROS/RNS production and NF-κB activity imaging results. The increase in RNS/ROS production was accompanied with a pronounced PMN recruitment within the inflamed ears (Fig. 3a, b).

**Fig. 4:**
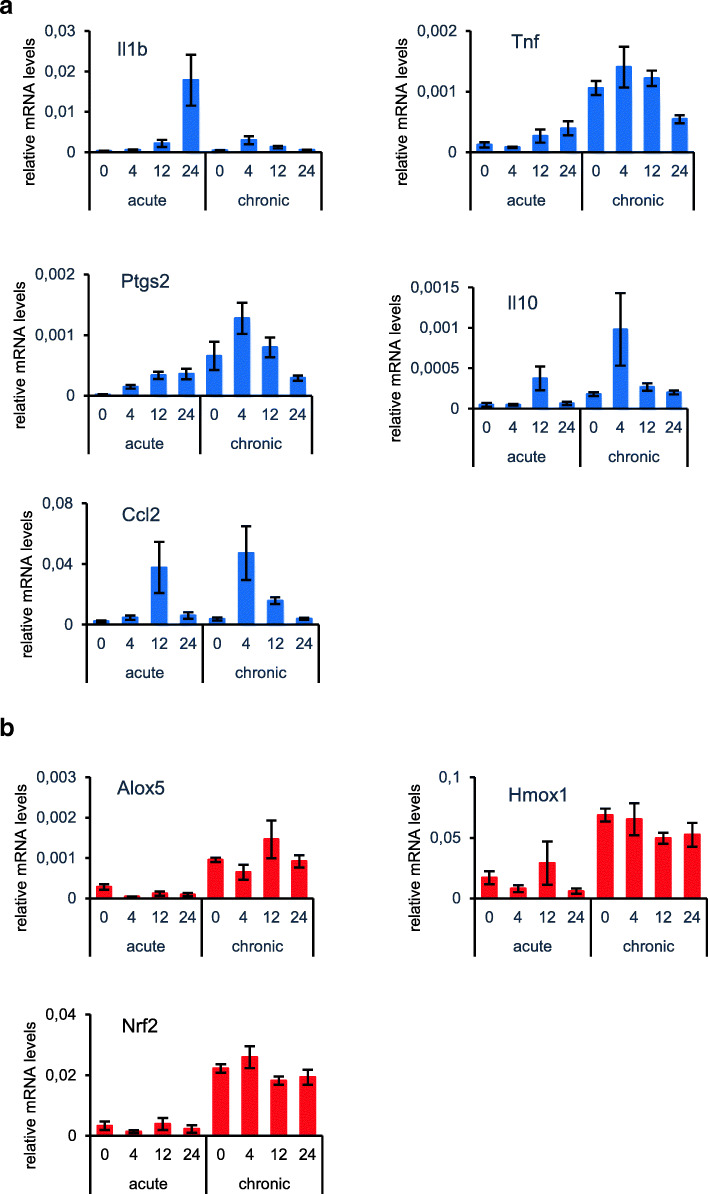
qPCR analysis of NF-κB- and ROS-/RNS-driven genes of ((a) naïve mice (0 h), inflamed ears with (a) acute cutaneous DTHR 4 h, 12 h, and 24 h after the 1st TNCB ear challenge and (b) chronic cutaneous DTHR before (48 h after the 4th TNCB ear challenge) 4 h, 12 h, and 24 h after the 5th TNCB ear challenge (*n* = 4). Data are represented as mean +/- SEM.

Next, we focused on the gene expression patterns in healthy and inflamed ears with acute and chronic cutaneous DTHR. A remarkable increase in the expression of NF-κB-driven genes encoding II1b, TNF, and Ptgs2 (COX-2) became evident 12 h after the 1st TNCB ear challenge. In line with the NF-κB imaging results, induction of pro-inflammatory gene expression peaked after 24 h. Ccl2 and Il10 mRNA expression reached a maximum as early as 12 h after the 1st TNCB ear challenge (Fig. 4a). In chronic DTHR, all investigated NF-κB-driven genes peaked already 4 h after 5th TNCB ear challenge (Fig. 4a), which coincided with the strongly enhanced NF-κB signal in the inflamed ears of the luciferase-reporter mice peaking at 12 h (Fig. 2b). The mRNA expression levels of ROS-inducible genes did not follow the *in vivo* dynamics of ROS production during acute and chronic DTHR (Fig. 4b). Nevertheless, an increase in mRNA expression of ROS-regulated proteins (Nrf2, Hmox1 (HO-1), and Alox5 (LOX-5)) was evident in chronic cutaneous DTHR when compared with the acute phase (Fig. 4b).

### Impact of NAC Treatment on ROS Production and NF-κB Activity

Next, we analyzed the differential effects of NAC treatment on ROS/RNS production expression and NF-κB activity during acute and chronic DTHR. In agreement with our recent experiments [20], NAC treatment significantly suppressed TNCB-induced ear-swelling responses during acute and chronic DTHR when compared with those of sham-treated mice.

**Fig. 5:**
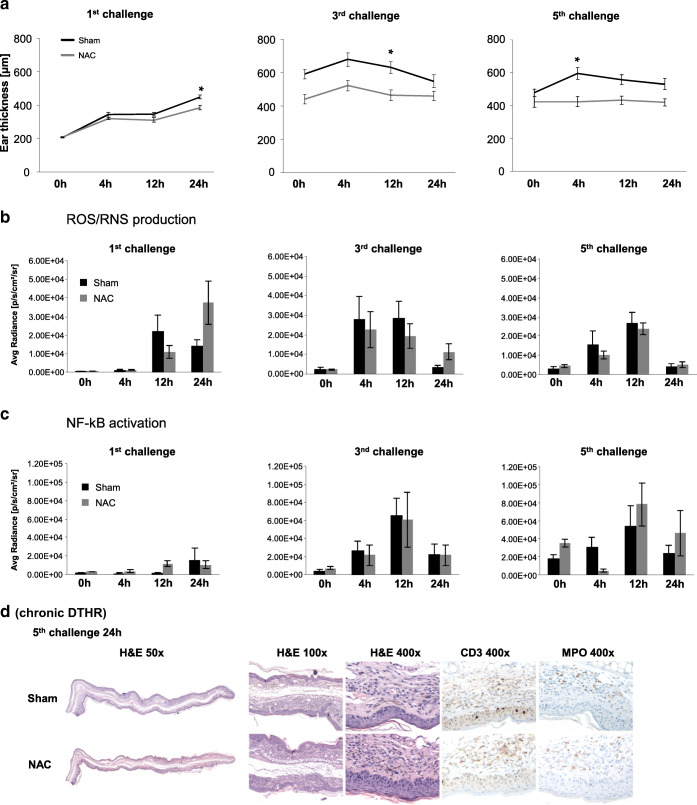
Impact of NAC-treatment on ROS/RNS production and NF-κB activity (NF-κB-luciferase-reporter mice: NAC treatment *n* = 5, sham treatment *n* = 4; L-012: NAC treatment *n* = 5, sham treatment *n* = 5). (A) Time course of the ear thickness 0–24 h after the 1st, 3rd, and 5th TNCB ear challenges. (B) Temporal dynamics of ROS/RNS production in inflamed ears of NAC-treated or sham-treated mice with acute, early chronic, and chronic cutaneous DTHR. (C) Temporal dynamics of NF-κB activation in inflamed ears of NAC-treated or sham-treated mice with acute, early chronic, and chronic cutaneous DTHR. Data are presented as mean ± SEM. (D) H&E histology and MPO and CD3 IHC 24 h after the 5th TNCB ear challenge (chronic cutaneous DTHR; *n* = 4).

Despite the reported significant anti-inflammatory effect of NAC (Fig. 5a), its impact on ROS/RNS production and NF-κB activity was heterogeneous. Maximum ROS/RNS production in inflamed ears was reached at 12 h after the 1st challenge in sham-treated mice and at 24 h in NAC-treated mice, whereas NF-κB expression peaked at 24 h in sham-treated mice and at 12 h in NAC-treated mice (Fig. 5b and c). Intriguingly, ROS production was higher in NAC-treated mice at 24 h following the 1st TNCB ear challenge than in sham-treated mice.

After the 3rd TNCB ear challenge, which corresponded to early chronic DTHR, ROS/RNS production in inflamed ears of NAC-treated mice was normal at 4 h and 12 h post challenge, but it was 3-fold higher at 24 h post challenge compared with that of sham-treated mice. In contrast, NF-κB activity in inflamed ears of NAC-treated mice was completely unchanged 4–24 h after TNCB ear challenge when compared with that of sham-treated mice (Fig. 5b and c).

After the 5th challenge, during chronic cutaneous DTHR, we observed almost no reduction in ROS production in inflamed ears of NAC-treated mice when compared with that of inflamed ears of sham-treated mice (Fig. 5b). In contrast, NF-κB activity was strongly reduced 4 h after the 5th TNCB ear challenge as a consequence of NAC treatment and was marginally elevated after 12 h and 24 h in comparison with that of sham-treated mice (Fig. 5c).

Histological analysis of inflamed ears 24 h after the 5th TNCB ear challenge, a time point when the ear-swelling response in NAC-treatment mice was only moderately reduced compared with the sham-treatment (Fig. 5a), revealed acanthosis with the presence of intra-epidermal PMNs and focal hyperkeratosis as well as edema and dilated blood and lymphatic vessels in the dermis in both experimental groups (Fig. 5c; Suppl. Fig. 3—see ESM). The inflammatory infiltrate was focal and mild in the H&E staining, while CD3 and MPO IHC did not reveal significant differences between the two experimental groups (Fig. 5d), correlating well to our *in vivo* L-012 imaging results (Fig. 5c).

## Discussion

In this study, we noninvasively evaluated the temporal dynamics of ROS/RNS production and NF-κB activation during acute and chronic cutaneous DTHR *in vivo*. During acute cutaneous DTHR, both ROS/RNS production and NF-κB activation were strongly increased 12 h after the first TNCB challenge and reached a maximum after 24 h. During early chronic cutaneous DTHR, ROS/RNS production and NF-κB activation peaked simultaneously 12 h after the third repetitive TNCB ear challenge. While in chronic cutaneous DTHR, after five repetitive TNCB ear challenge, we were able to measure strongly elevated ROS/RNS production and NF-κB activation as early as 4 h (Fig. 2a and b).

Both ROS/RNS production and NF-κB have been extensively investigated *in vitro* but have hardly been investigated *in vivo*. Sustained ROS stress *in vitro* causes proteasome inactivation by 50–80 % and therefore less degradation of the inhibitory I-κBα protein, resulting in suppressed NF-κB activation [26].

A characteristic feature of the NF-κB signaling pathway is that immediate responses to various stimuli are feasible, as the transcription factors are stored in an inactive state in the cytoplasm. In general, two distinct NF-κB signaling pathways that result in two different NF-κB-dependent gene expression patterns have been described (See Suppl. 1 Discussion in ESM).

Among the NF-κB-driven genes are multiple antioxidant and ROS-/RNS-promoting gene targets, but NF-κB itself also heavily influences ROS/RNS expression [27]. ROS/RNS measurements are especially challenging *in vivo*. In our study, we confirmed the feasibility of noninvasively determining ROS/RNS production *in vivo* and longitudinally monitoring the changes in ROS/RNS production during progression from acute to chronic cutaneous DTHR. We employed the chemiluminescent probe L-012, which has been extensively used in several *in vitro* studies, but the use of L-012 in *in vivo* studies is rare. Kielland et al. used L-012 for *in vivo* studies in experimental LPS-induced acute systemic inflammation, PMA-induced inflammation of the ear, and collagen-induced arthritis [10].

Another established chemiluminescent probe is lucigenin. Tseng et al. demonstrated that the lucigenin chemiluminescent signal is independent of MPO but requires NADPH oxidase (Phox) activity in macrophages, while the luminol chemiluminescent signal largely depends on MPO expression by PMNs [28].

In our experiments, the measured L-012 signal intensity in inflamed ears with acute cutaneous DTHR closely followed the infiltration of MPO^+^ PMNs, both peaking 24 h after the 1st TNCB ear challenge (Figs. 1a,3a). In chronic DTHR, a slightly elevated L-012 signal intensity was detectable before the 5th TNCB ear challenge (0 h), 48 h after the 4th TNCB ear challenge, as a consequence of the already established skin inflammation and the presence of MPO^+^ PMNs (Figs. 2a, 3b). The L-012 signal peaked already after 4 h, coinciding with an increased number of MPO^+^ PMNs in the dermis. Thereafter, the L-012 signal decreased until 24 h after the 5th TNCB ear challenge, while the number of MPO^+^ PMNs remained constant (Figs. 2a, 3b). Interestingly, the ROS/RNS production values in chronic cutaneous DTHR were lower than in the acute phase of inflammation (Figs. 1a, 2a). Analysis of the genomic expression of ROS-/RNS-driven antioxidative proteins revealed elevated expression patterns in chronic cutaneous DTHR when compared with the acute phase (Fig. 4b). This might explain why the ROS/RNS production values, determined by L-012 optical imaging, were lower in chronic when compared with acute cutaneous DTHR as antioxidative proteins need time to be produced and expressed [29–31]. The mRNA expression of ROS-/RNS-inducible genes is a part of the cellular response to oxidative stress, the regulation of which is slower and maybe more complex than the response to NF-κB activation which reached similarly high signal intensity levels in NF-κB-reporter mice with acute and chronic cutaneous DTHR (Fig. 1b, Fig. 2b). In addition, it has to be taken in account that the immune cell infiltrate in acute and chronic cutaneous DTHR differs significantly (Fig. 3a and b). Thus, the NF-κB activity in acute DTHR might be mainly related to the affected keratinocytes and the infiltration of PMNs, whereas in the chronic phase, the immune cell infiltrate is more heterogeneous and composed of PMNs, T cells, B cells, and macrophages. For instance, the strongly enhanced Il1b mRNA expression in acute DTHR most likely originates from keratinocytes, whereas the immune cell infiltrate might be mainly responsible for the elevated TNF expression (Fig. 4a and b).

We used two different noninvasive *in vivo* tools to measure ROS/RNS production and NF-κB activation and to longitudinally monitor the antioxidative and NF-κB signaling inhibiting properties of NAC during different stages of cutaneous DTHR. In accordance with our previously published data [20], NAC was highly efficient in suppressing ear-swelling responses during acute and chronic cutaneous DTHR due to its antioxidative and NF-κB-inhibiting effects (Fig. 5a).

Surprisingly, we were not able to identify a clear trend when comparing the measured *in vivo* ROS/RNS production and NF-κB activity in inflamed ears of NAC- and sham-treated mice (Fig. 5b and c). At the later time points, especially 24 h after the 1st (acute DTHR) and 3rd (early chronic DTHR) TNCB ear challenges, we observed enhanced ROS/RNS production in inflamed ears of NAC-treated mice, but at the earlier time points, we saw a rather reduced ROS/RNS production when compared with that of the sham-treatment (Fig. 5b). Thus, NAC treatment might mainly change the temporal dynamics of ROS/RNS production. Despite the indisputable therapeutic effect of NAC (Fig. 5a, right graph), we measured almost no NAC treatment-induced effect on ROS/RNS production in inflamed ears with chronic cutaneous DTHR 24 h after the 5th TNCB ear challenge (Fig. 5b, right graph). NAC treatment was effective during acute, and early chronic and chronic cutaneous DTHR. We observed an elevated baseline (0 h) NF-κB activity level, strongly reduced levels at 4 h after the 5th TNCB ear challenge, and rather enhanced activity after 12 h and 24 h (Fig. 5c).

Despite its widespread use, e.g., as a mucolytic agent or antidote for acetaminophen intoxication, many of the complex and controversial effects induced by NAC are still under debate (See Suppl. 2 Discussion in ESM).

## Conclusions

The temporal dynamics of ROS/RNS production and NF-κB activity during the progression from acute to chronic T cell–driven cutaneous DTHR, and during a ROS/RNS and NF-κB activity targeting treatment approach, can be noninvasively monitored *in vivo*. Our study revealed that ROS/RNS production and also NF-κB activity are highly dynamic and are part of a complex interplay between immune cell migration and gene expression. Determination of the temporal dynamics of ROS/RNS production and NF-κB activity may open a new avenue to understand, characterize, and noninvasively monitor inflammatory responses *in vivo*; therefore, it might enable the improvement of therapeutic interventions. This knowledge could be a prerequisite for the identification of the therapeutic windows for innovative treatment approaches in other types of DTHR, such as psoriasis or rheumatoid arthritis.

## Electronic supplementary material

ESM 1 (PDF 1.41 mb)
