# Supplementary material for: Temporal Dynamics of Reactive Oxygen and Nitrogen Species and NF-κB Activation During Acute and Chronic T Cell–Driven Inflammation
Source: Mol Imaging Biol. 2019 Sep 3;22(3):504–14. doi: 10.1007/s11307-019-01412-8 (PMC7250960; doi:10.1007/s11307-019-01412-8)
Supplement: Supplementary file 1 — (PDF 1.41 mb) [file 11307_2019_1412_MOESM1_ESM.pdf]

Electronic Supplementary Material

Differential Temporal Dynamics of Reactive Oxygen and Nitrogen Species and NF-κB Activation During Acute and Chronic T Cell-Driven Inflammation

Journal: Molecular Imaging and Biology

Johannes Schwenck[1,2], Roman Mehling[1], Wolfgang M. Thaiss[1,3], Daniela Kramer[4], Irene Gonzalez Menendez[5], Hasan Halit Öz[6], Dominik Hartl[6], Klaus Schulze-Osthoff[4,7], Stephan Hailfinger[4], Kamran Ghoreschi[8], Leticia Quintanilla-Martinez[5], Harald Carlsen[9], Martin Röcken[7,10], Bernd J. Pichler[1,7], Manfred Kneilling[1,10*]

[1]Werner Siemens Imaging Center, Department of Preclinical Imaging and Radiopharmacy, Eberhard Karls University, 72076 Tübingen, Germany

[2]Department of Nuclear Medicine, Eberhard Karls University, 72076 Tübingen, Germany

[3]Department of Diagnostic and Interventional Radiology, Eberhard Karls University, 72076 Tübingen, Germany

[4]Interfaculty Institute of Biochemistry, Eberhard Karls University of Tübingen, Germany

[5]Department of Pathology, Eberhard Karls University, 72076 Tübingen, Germany

[6]Department of Pediatrics I, Eberhard Karls University, 72076 Tübingen, Germany

[7]German Cancer Consortium (DKTK) and German Cancer Research Center, 69120 Heidelberg, Germany

[8]Department of Dermatology, Venereology and Allergology, Charité – Universitätsmedizin Berlin, 10117 Berlin, Germany

[9]Department of Chemistry, Biotechnology and Food Science, Norwegian University of Life Sciences, 1432, Ås, Norway

[10]Department of Dermatology, Eberhard Karls University, 72076 Tübingen, Germany

* Corresponding author email: manfred.kneilling@med.uni-tuebingen.de
telephone: +49-7071-29-86870
fax: +49-7071-29-4451

Short title: *In vivo* imaging of ROS production and NF-κB activation in inflammation

Materials and Methods

Animal experiments

Mice were sensitized by topical application of TNCB (5% TNCB; 80 µl dissolved in a 4:1 mixture of acetone/Miglyol 812; Sasol, Witten, Germany) to the abdomen and challenged on the right ear at day 7 (1% TNCB; 20 µl dissolved in a 9:1 mixture of acetone/Miglyol 812) to elicit acute DTHR. To induce chronic DTHR, mice were challenged every 48 h, for up to 5 times. Ear swelling was quantified by measuring the ear thickness with a micrometer (Kroeplin, Schlüchtern, Germany) before TNCB ear challenge and 4 h to 24 h afterwards. All measurements of the challenged right ears were compared to the nonchallenged left ears of the mice.

Optical imaging

OI measurements were performed 5 min after injection of 100 µl L-012 solution (5 mg/ml) [1]. During optical imaging, mice were anesthetized by inhalation of isoflurane-O_2 (1.5% Forane, Abbott GmbH, Wiesbaden, Germany) and placed on a heating pad to maintain body temperature between 36°C and 37°C. To measure the whole upper surface area of the mouse ears, the ears were carefully fixed on a flat black plate by a nylon thread. Regions of interest (ROIs) were drawn on the right and left ears, enabling a semiquantitative analysis of the detected bioluminescence and chemiluminescence average radiance [p/s/cm^2/sr] as previously described [1-2]. Image analysis was performed using Living Image Software (Perkin Elmer).

RNA extraction and gene expression analysis

Ear samples of the treated mice were sheared in Qiazol (79306, Qiagen) and total RNA was isolated in accordance with the manufacture`s protocol. Genomic DNA was removed by DNAse I digestion (EN0523, Thermo Fisher), followed by cDNA

synthesis using oligo(dT) primer (SO132, Thermo Fisher) and Revert Aid reverse transcriptase (EP0441, Thermo Fisher). The expression of the indicated genes was quantified using the Green Master mix (M3023, Genaxxon) and self-designed primers (Suppl Table 1). PCR conditions were as follows: Initial denaturation 15 min at 95°C, followed by 45 cycles of 95°C for 15 s and 60°C for 45 s. Relative mRNA levels were calculated by normalization to the reference gene *Actin* using the $2^{-\Delta\Delta CT}$ method.

Histopathology

Tissue samples were fixed in 4% formalin and subsequently paraffin embedded. For histology 3-5 µm-thick sections were cut and stained with haematoxylin and eosin (H&E). Immunohistochemistry was performed on an automated immunostainer (Ventana Medical Systems, Inc.) according to the company’s protocols for open procedures with slight modifications. The slides were stained with the antibodies CD3 (Clone SP7, DCS Innovative Diagnostik-Systeme GmbH u. Co. KG, Hamburg, Germany) and MPO (Anti-Myeoloperoxidase Ab-1, Lab Vision UK, Ltd., Newmarket, Suffolk). Appropriate positive and negative controls were used to confirm the adequacy of the staining. Photomicrographic images were acquired with an Axioskop 2 plus Zeiss microscope equipped with a Jenoptik (Laser Optik System, Jena, Germany) ProgRes C10 plus camera and software. The epidermal inflammation score was based on the presence and number of epidermal abscesses and crusts per section (0 = no damage, 1 = presence of abscesses, 2 = between 1 and 5 crusts, 3 = between 6 and 10 crusts, 4 = more than 11 crusts). A semiquantative analysis of dermal inflammation was also performed (“-“ = no inflammatory infiltrate, “+” = minimal inflammatory cells, “++” = mild inflammation, “+++” = moderate inflammation, “++++” = severe presence of inflammatory cells).

Statistical analysis

A paired, two tailed Student's t test was used to compare the peak of the optical imaging signal and the baseline signal **(**Fig. 1a and b; Fig. 2a and b**)**. Unpaired, two-tailed Student's t test was utilized to compare the relative changes of ROS production and NF-κB activation after the first TNCB challenge as well as after the third and the fifth TNCB challenge (Fig. 1c and 2c). Differences in ear thickness, L-012 signal intensities or NF-κB activation signal intensities (NF-κB reporter mice) between NAC- and sham-treated mice were examined by unpaired, two-tailed Student's t test (Fig. 3a - c). P values below 0.05 were considered statistically significant. Quantitative data are reported as the mean and standard error of the mean (± 1 SEM).

Suppl. Discussion 1

The heterodimer of the proteins p50 and RelA (p65) form the canonical NF-κB signaling pathway, whereas p52 and RelB are driving the alternative (non-canonical) NF-κB signaling pathway [3-5]. While the canonical NF-κB signaling pathway is faster and more involved in unspecific innate immune responses, the slower alternative pathway is associated with adaptive immune responses and developmental processes [6-9]. Knockout experiments revealed that mice deficient in alternative NF-κB signaling are unable to develop an adequate adaptive immune response against viral infections [10]. However, mice deficient in classical NF-κB signaling are more sensitive to bacterial infections [11]. Together both, the canonical and alternative NF-κB signaling pathway, represent an interdependent, highly complex regulation system that are influencing each other by expression of NF-κB-monomers, phosphorylation or cleavage of pro-forms [12-16]. Both NF-κB signaling pathways are interacting with hundreds of gene loci due to a distinct palindromic DNA sequence [17]. Physiologically NF-κB signaling is involved in embryonal development as well as in immune reactions. The relevance of functional NF-κB signaling is underlined by multiple diseases directly caused by genetic malfunctions of NF-κB proteins leading mostly to immune defects or developmental disorders [17]. Beyond that, the onsets of a broad range of diseases are caused by cellular responses transmitted by NF-κB signaling, including cancer or neurodegeneration [18-19].

ROS species interfere with both the canonical as well as the noncanonical NF-κB signaling pathways and are believed to be major regulators of NF-κB-mediated cell responses [20-24]. How ROS/RNS interact with NF-κB pathways has not yet been fully elucidated. *In vitro* experiments show that cysteines of NF-κB signaling molecules can be oxidized by ROS/RNS, resulting in inactivation of NF-κB signaling

molecules. This inactivation can in turn lead to activation or inactivation of the NF-κB signaling pathway depending on the negative or positive regulatory function of the oxidized NF-κB signaling molecule. The oxidation can be reversed by antioxidative glutathione [21]. Furthermore, the oxidation of a specific cysteine within the p50 molecule can lead to the inhibition of its DNA binding and therefore to reduced NF-κB signaling. In contrast, mitochondrial ROS are able to enhance NF-κB signaling [25]. Modulatory signaling pathways upstream of NF-κB (e.g., TNF signaling by ROS) are another mode of interaction [21, 26].

Suppl. Discussion 2

It has been demonstrated that NAC can increase the ability of the NF-κB transcription factor subunit p65 to bind to DNA [27]. Previously, we reported a suppressive effect of NAC treatment on matrix metalloproteinase activity and angiogenesis [2]. The p38 MAP kinase pathway represents another major ROS-sensitive signaling pathway that is involved in establishing and maintaining inflammatory responses [28-32]. NAC effectively inhibits p38 MAP kinase signaling in T cells and dendritic cells [33-34]. In our experiments, we observed a nonuniform effect of NAC treatment on *in vivo* ROS/RNS production and NF-κB activity despite the clear anti-inflammatory effect demonstrated by the reduced ear swelling responses, possibly due to interactions with other pathways.

Supplementary References

1. Fuchs K, Kuehn A, Mahling M, et al. (2017) In Vivo Hypoxia PET Imaging Quantifies the Severity of Arthritic Joint Inflammation in Line with Overexpression of Hypoxia-Inducible Factor and Enhanced Reactive Oxygen Species Generation. J Nucl Med 58:853-860.
2. Schwenck J, Griessinger CM, Fuchs K, et al. (2014) In vivo optical imaging of matrix metalloproteinase activity detects acute and chronic contact hypersensitivity reactions and enables monitoring of the antiinflammatory effects of N-acetylcysteine. Mol Imaging 13.
3. Perkins ND (2007) Integrating cell-signalling pathways with NF-kappaB and IKK function. Nat Rev Mol Cell Biol 8:49-62.
4. Hayden MS, Ghosh S (2004) Signaling to NF-kappaB. Genes Dev 18:2195-2224.
5. Hayden MS, Ghosh S (2008) Shared principles in NF-kappaB signaling. Cell 132:344-362.
6. Bonizzi G, Karin M (2004) The two NF-kappaB activation pathways and their role in innate and adaptive immunity. Trends Immunol 25:280-288.
7. Brown KD, Claudio E, Siebenlist U (2008) The roles of the classical and alternative nuclear factor-kappaB pathways: potential implications for autoimmunity and rheumatoid arthritis. Arthritis Res Ther 10:212.
8. Sun SC (2011) Non-canonical NF-kappaB signaling pathway. Cell Res 21:71-85.
9. Luftig M, Yasui T, Soni V, et al. (2004) Epstein-Barr virus latent infection membrane protein 1 TRAF-binding site induces NIK/IKK alpha-dependent noncanonical NF-kappaB activation. Proc Natl Acad Sci U S A 101:141-146.
10. Droebner K, Klein B, Paxian S, Schmid R, Stitz L, Planz O (2010) The alternative NF-kappaB signalling pathway is a prerequisite for an appropriate immune response against lymphocytic choriomeningitis virus infection. Viral Immunol 23:295-308.
11. Sha WC, Liou HC, Tuomanen EI, Baltimore D (1995) Targeted disruption of the p50 subunit of NF-kappa B leads to multifocal defects in immune responses. Cell 80:321-330.
12. O'Dea E, Hoffmann A (2010) The regulatory logic of the NF-kappaB signaling system. Cold Spring Harb Perspect Biol 2:a000216.
13. Shih VF, Tsui R, Caldwell A, Hoffmann A (2011) A single NFkappaB system for both canonical and non-canonical signaling. Cell Res 21:86-102.
14. Basak S, Shih VF, Hoffmann A (2008) Generation and activation of multiple dimeric transcription factors within the NF-kappaB signaling system. Mol Cell Biol 28:3139-3150.
15. Rao P, Hayden MS, Long M, et al. (2010) IkappaBbeta acts to inhibit and activate gene expression during the inflammatory response. Nature 466:1115-1119.
16. Madge LA, May MJ (2010) Classical NF-kappaB activation negatively regulates noncanonical NF-kappaB-dependent CXCL12 expression. J Biol Chem 285:38069-38077.
17. Zhang Q, Lenardo MJ, Baltimore D (2017) 30 Years of NF-kappaB: A Blossoming of Relevance to Human Pathobiology. Cell 168:37-57.
18. Karin M (2009) NF-kappaB as a critical link between inflammation and cancer. Cold Spring Harb Perspect Biol 1:a000141.
19. Mincheva-Tasheva S, Soler RM (2013) NF-kappaB signaling pathways: role in nervous system physiology and pathology. Neuroscientist 19:175-194.

20. D'Autreaux B, Toledano MB (2007) ROS as signalling molecules: mechanisms that generate specificity in ROS homeostasis. Nat Rev Mol Cell Biol 8:813-824.
21. Morgan MJ, Liu ZG (2011) Crosstalk of reactive oxygen species and NF-kappaB signaling. Cell Res 21:103-115.
22. Dolado I, Swat A, Ajenjo N, De Vita G, Cuadrado A, Nebreda AR (2007) p38alpha MAP kinase as a sensor of reactive oxygen species in tumorigenesis. Cancer Cell 11:191-205.
23. Torres M, Forman HJ (2003) Redox signaling and the MAP kinase pathways. Biofactors 17:287-296.
24. Michiels C, Minet E, Mottet D, Raes M (2002) Regulation of gene expression by oxygen: NF-kappaB and HIF-1, two extremes. Free Radic Biol Med 33:1231-1242.
25. Hughes G, Murphy MP, Ledgerwood EC (2005) Mitochondrial reactive oxygen species regulate the temporal activation of nuclear factor kappaB to modulate tumour necrosis factor-induced apoptosis: evidence from mitochondria-targeted antioxidants. Biochem J 389:83-89.
26. Lingappan K (2018) NF-kappaB in Oxidative Stress. Curr Opin Toxicol 7:81-86.
27. Liu J, Yoshida Y, Yamashita U (2008) DNA-binding activity of NF-kappaB and phosphorylation of p65 are induced by N-acetylcysteine through phosphatidylinositol (PI) 3-kinase. Mol Immunol 45:3984-3989.
28. Kamata H, Manabe T, Kakuta J, Oka S, Hirata H (2002) Multiple redox regulation of the cellular signaling system linked to AP-1 and NFkappaB: effects of N-acetylcysteine and H2O2 on the receptor tyrosine kinases, the MAP kinase cascade, and IkappaB kinases. Ann N Y Acad Sci 973:419-422.
29. Wuyts WA, Vanaudenaerde BM, Dupont LJ, Demedts MG, Verleden GM (2003) N-acetylcysteine reduces chemokine release via inhibition of p38 MAPK in human airway smooth muscle cells. Eur Respir J 22:43-49.
30. Hashimoto S, Gon Y, Matsumoto K, Takeshita I, Horie T (2001) N-acetylcysteine attenuates TNF-alpha-induced p38 MAP kinase activation and p38 MAP kinase-mediated IL-8 production by human pulmonary vascular endothelial cells. Br J Pharmacol 132:270-276.
31. Dong C, Davis RJ, Flavell RA (2002) MAP kinases in the immune response. Annu Rev Immunol 20:55-72.
32. Ashwell JD (2006) The many paths to p38 mitogen-activated protein kinase activation in the immune system. Nat Rev Immunol 6:532-540.
33. Verhasselt V, Vanden Berghe W, Vanderheyde N, Willems F, Haegeman G, Goldman M (1999) N-acetyl-L-cysteine inhibits primary human T cell responses at the dendritic cell level: association with NF-kappaB inhibition. J Immunol 162:2569-2574.
34. Bruchhausen S, Zahn S, Valk E, Knop J, Becker D (2003) Thiol antioxidants block the activation of antigen-presenting cells by contact sensitizers. J Invest Dermatol 121:1039-1044.

Suppl. Table 1

gene	forward primer	reverse primer
Actin	AGGAGTACGATGAGTCCGGC	GGTGTAAAACGCAGCTCAGTA
Alox5	TGTACACACCAGTTCCTGGC	GTTTGGTTGAGCTGGATGGC
Ccl2	CTGGAGCATCCACGTGTTGG	CCCATTCCTTCTTGGGGTCAG
Hmox1	TGACACCTGAGGTCAAGCAC	AAGTGACGCCATCTGTGAGG
Il10	GCATTTGAATTCCCTGGGTGAG	CATGGCCTTGTAGACACCTTGG
Il1b	AGCTGAAAGCTCTCCACCTC	GCTTGGGATCCACACTCTCC
Nrf2	TAGTTCTCCGCTGCTCGGAC	TGTCTTGCCTCCAAAGGATGTC
Ptgs2	AGCAGATGACTGCCCAACTC	GGAAGCTCCTTATTTCCCTTCAC
Tnf	AAGTTCCCAAATGGCCTCCC	TTGCTACGACGTGGGCTAC

Suppl. Table 2

1st challenge

	score	MPO	CD3
**naive**	0	-	-
**naive**	0	-	-
**naive**	0	-	-
**naive**	0	-	-
**4 h**	0	-	-
**4 h**	0	-	-
**4 h**	0	+	+
**4 h**	0	+	+
**12 h**	0	+	+
**12 h**	0	++	+
**12 h**	0	++	+
**12 h**	0	+++	+
**24 h**	1	+	+
**24 h**	2	++++	+
**24 h**	2	++++	++
**24 h**	3	++++	+

5th challenge

	score	MPO	CD3
**0 h**	0	+++	+
**0 h**	1	+	++
**0 h**	2	++	+
**0 h**	2	+	++
**4 h**	2	++	+
**4 h**	2	+	+
**4 h**	0	+++	++
**4 h**	2	+++	+++
**12 h**	2	+++	+++
**12 h**	2	++	++
**12 h**	2	++	+++
**24 h**	2	+++	+++
**24 h**	2	+++	+++
**24 h**	1	++	+++
**24 h**	2	++	++

Results of the histological scoring: Epidermal inflammation score was based on the presence and number of epidermal abscesses and crusts per section (0 = no damage, 1 = presence of abscesses, 2 = between 1 and 5 crusts, 3 = between 6 and 10 crusts, 4 = more than 11 crusts). IHC was analysed by a semiquantative analysis of dermal inflammation (“-“ = no inflammatory infiltrate, “+” = minimal inflammatory cells, “++” = mild inflammation, “+++” = moderate inflammation, “++++” = severe presence of inflammatory cells). (n = 4; only 12 h after the 5th TNCB ear challenge: n = 3).

Suppl. Fig. 1

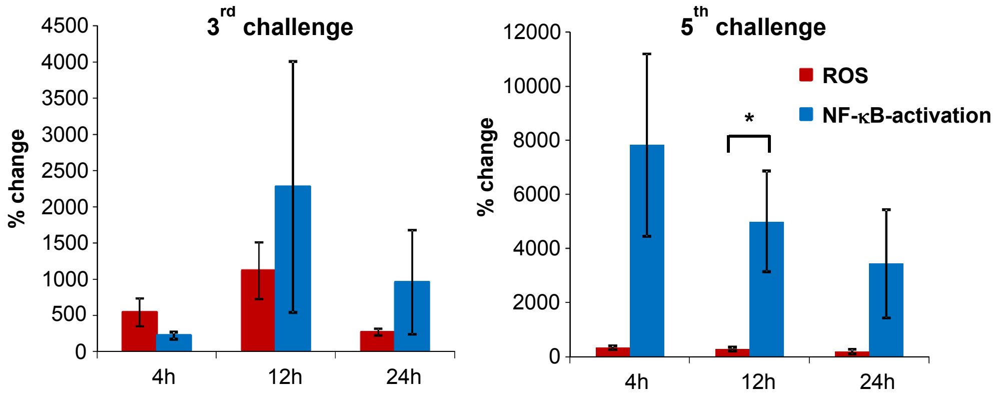

When calculating the relative change in signal intensity within the course of the 3rd or 5th TNCB ear challenge, using 0 h of the 3rd or 5th challenge as baseline, the relative increase in NF-κB activity 12 h and 48 h after the 3rd TNCB ear challenge and especially 4 h, 12 h and 24 h after the 5th TNCB ear challenge was impressively higher whereas the relative increase in ROS/RNS production was moderate. 12 h after the 5th TNCB challenge the relative change in NF-κB activation was significantly higher compared to the relative change in ROS/RNS production (unpaired, two-tailed Student's *t*-test). Data are presented as the mean ± SEM.

Suppl. Fig. 2

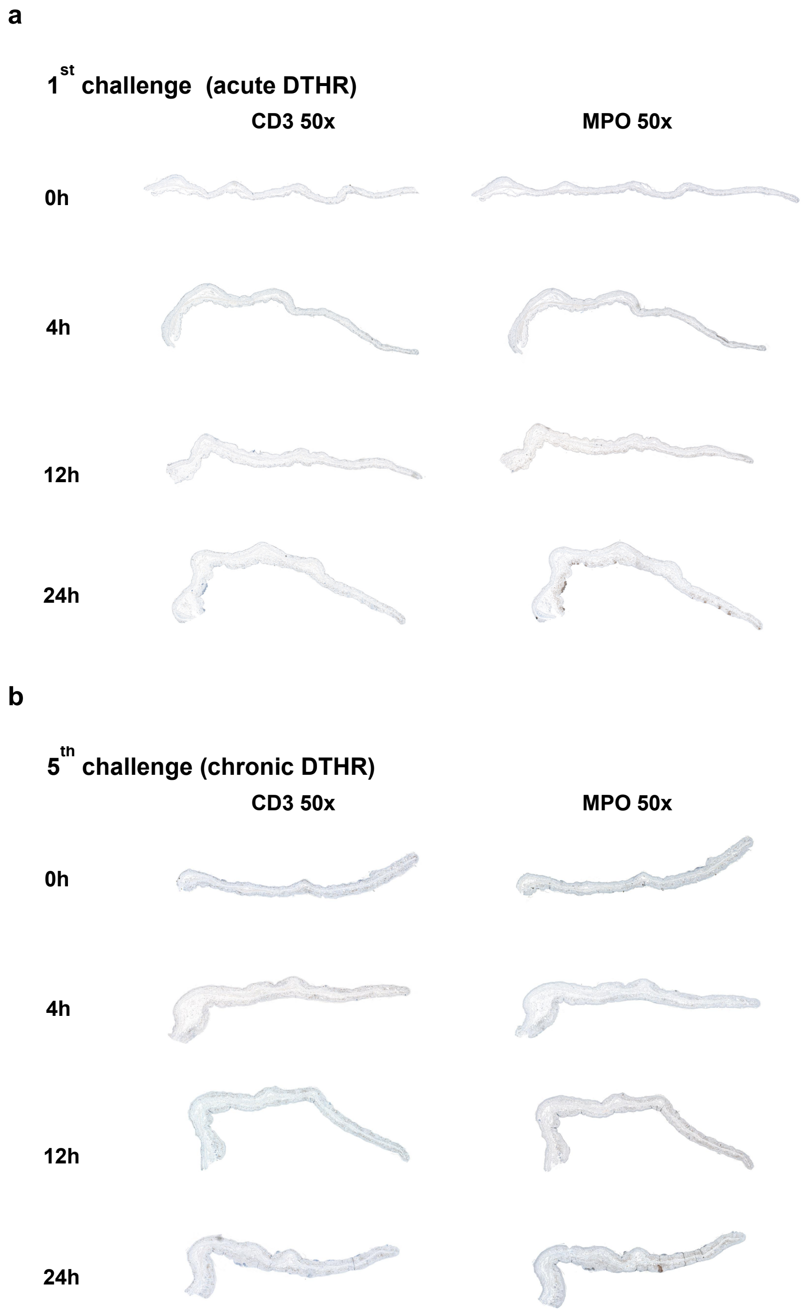

Corresponding images with 50x magnification of the CD3 and MPO IHC from **(a)** naïve mice (0 h), inflamed ears with **(a)** acute cutaneous DTHR 4 h, 12h and 24 h after the 1st TNCB ear challenge and **(b)** chronic cutaneous DTHR before (48 h after the 4th TNCB ear challenge) 4 h, 12h and 24 h after the 5th TNCB ear challenge (n = 4; only 12 h after the 1st TNCB ear challenge: n = 3).

Suppl. Fig. 3

5th challenge 24h (chronic DTHR)

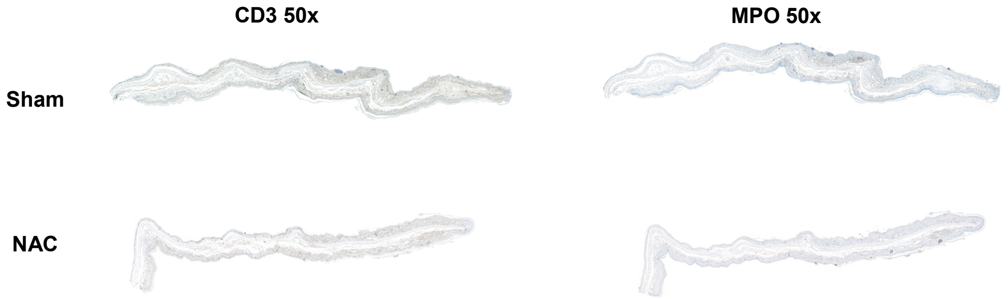

Corresponding images with 50x magnification of the CD3 and MPO IHC of NAC- and sham treated mice 24 h after the last TNCB ear challenge. CD3 and MPO IHC did not reveal significant differences between the two experimental groups.
